# Effects of oral, oronasal, and oronasal breathing with a decongested nose during incremental maximal exercise testing of well-trained endurance athletes: a randomized cross-over study

**DOI:** 10.3389/fphys.2025.1654725

**Published:** 2025-09-24

**Authors:** J. Bergqvist, F. Reite, F. Edin, L. Schiöler, M. Börjesson, S. Steinsvåg, S. Pettersson, J. Hellgren

**Affiliations:** ^1^ Department of Respiratory Medicine and Allergology, Sahlgrenska University Hospital, Gothenburg, Sweden; ^2^ Centre for Sleep and Wake Disorders, The Sahlgrenska Academy, University of Gothenburg, Gothenburg, Sweden; ^3^ Department Of Otolaryngology, Head and Neck Surgery, Sørlandet Hospital, Kristiansand, Norway; ^4^ Centre for Health and Performance, Department of Food and Nutrition, and Sport Science, University of Gothenburg, Gothenburg, Sweden; ^5^ Occupational and Environmental Medicine, School of Public Health and Community Medicine, The Sahlgrenska Academy, University of Gothenburg, Gothenburg, Sweden; ^6^ Department of Molecular and Clinical Medicine, The Sahlgrenska Academy, University of Gothenburg, Gothenburg, Sweden; ^7^ Center for lifestyle Intervention, Department of MGAÖ, Östra Hospital, Gothenburg, Sweden; ^8^ Department Of Otolaryngology, Head and Neck Surgery, Sørlandet Hospital and Haukeland University Hospital, University of Bergen, Bergen, Norway; ^9^ Department of Otorhinolaryngology, Head & Neck Surgery, Sahlgrenska University Hospital, Region Västra Götaland, Gothenburg, Sweden; ^10^ Department of Otorhinolaryngology, Institute of Clinical Sciences, The Sahlgrenska Academy, University of Gothenburg, Gothenburg, Sweden

**Keywords:** VO_2max_, lactate, nasal breathing, rhinomanometry, athlete

## Abstract

**Introduction:**

Nasal breathing is preferable for persons at rest and remains partially active during oronasal breathing in exercise. However, its potential contribution to performance–particularly in cases with a decongested nose–remains understudied in well-trained athletes. This study investigates whether nasal airflow during oronasal breathing influences performance in well-trained, endurance athletes. Specifically, we examine whether nasal decongestion during oronasal breathing enhances ventilatory efficiency and, thereby, improves time-to-exhaustion (TTE), maximal oxygen uptake ( $\dot{V}$ O_2max_), and maximum power output (W_max_), as compared to oral-only breathing.

**Methods:**

Twelve male, well-trained cyclists/triathlon athletes (mean $\dot{V}$ O_2max_, 67.2 ± 5.5 mL kg^-1^·min^-1^) with age range of 30.6 ± 8.7 years, were included. Two characterization tests were performed: 1) an incremental cycle test to determine $\dot{V}$ O_2max_ and W_max_; and 2) a familiarization trial of the experimental exercise protocol. The three experimental exercise trials consisted of five 6-min submaximal steady-state levels (50 W and 100 W at 50 rpm for the first two stages, followed by 40%, 58%, and 75% of the individual W_max_ at 80 rpm), concluding with a TTE test.

**Results:**

There were no significant differences between the three breathing modes (p > 0.05) in terms of the cardiopulmonary or performance parameters, including the rate of perceived exertion, respiratory frequency, mean minute ventilation, $\dot{V}$ O_2max_, and W_max_. Although not statistically significant (p > 0.05) TTE was 2.8% and 4.2% longer during oronasal and decongested oronasal breathing, respectively, as compared to oral-only breathing. The mean capillary blood lactate level was significantly (p < 0.05) lower immediately after and 3 min after the TTE test in the oral-only breathing condition (9.12 ± 2.20 mmol/L), as compared with the oronasal (9.83 ± 2.19 mmol/L, Cohen’s *d* = 0.43) and decongested-nose (9.81 ± 2.29 mmol/L, *d* = 0.41) conditions.

**Conclusion:**

Oral-only breathing is associated with a non-significant shorter TTE than oronasal breathing with or without nasal decongestion, although it results in significantly lower mean capillary blood lactate levels following maximal aerobic exercise. These findings suggest that a single, low-resistance oral breathing route reduces lactate accumulation under maximal effort, whereas oronasal breathing–particularly in the presence of nasal decongestion–may be more beneficial for sustaining endurance.

## 1 Introduction

Nasal breathing is the preferred breathing route in most humans during sleep and at rest, even though approximately two-thirds of the total airway resistance occurs in the anterior part of the nose (Haight and Cole, 1983). As ventilatory demand increases during exercise, the nasal breathing capacity is eventually exceeded, prompting a transition to oronasal breathing. Nevertheless, continued nasal breathing during exercise has been proposed to enhance performance (Walker et al., 2016), which could include improvements in maximal oxygen uptake ( $\dot{V}$ O_2max_) and maximal power output (W_max_). One physiologic explanation for this is exercise-induced reduction of nasal airway resistance, which is attributed to vasoconstriction of the nasal mucosa due to sympathetic activation (Forsyth et al., 1983).

While some mechanisms have been proposed, the empirical findings have been inconsistent. LaComb et al. compared nasal and oral breathing during graded exercise and found that while oral breathing produced larger respiratory and metabolic volumes, it did not necessarily improve ventilatory efficiency (LaComb et al., 2017). Meir et al. reported no significant differences in performance, perceived exertion, blood lactate, or ventilatory parameters in rugby players who were performing repeated, high-intensity shuttle runs with or without nasal occlusion (Meir et al., 2014). Similarly, Recinto and coworkers observed no significant differences in power output or performance between subjects with nasal and oral-only breathing in a Wingate anaerobic cycling test (Recinto et al., 2017).

A systematic review and meta-analysis concluded that external nasal stents did not significantly improve $\dot{V}$ O_2max_ during aerobic exercise in healthy individuals (Dinardi et al., 2021). In line with this, Niinimaa and colleagues estimated that the transition from nasal to oronasal breathing occurred at a mean workload of 105 W and a minute ventilation (VE) rate of 35 L/min, suggesting a physiologic ceiling for nasal airflow under increasing load (Niinimaa et al., 1980). However, this transition point varies significantly between individuals. Some persons maintain nasal breathing longer during submaximal workloads, while others shift earlier to oronasal breathing. Importantly, during oronasal breathing, the nasal airway remains open, as the airflows from both routes converge at the oropharynx. This means that the nasal airflow may still contribute meaningfully to total ventilation even after the shift from exclusively nasal breathing. It has been estimated that nasal breathing can contribute up to 61% of the total VE at 45 L/min, suggesting that it may remain relevant even at higher exercise intensities (Niinimaa et al., 1981). Still, this has not been thoroughly studied in well-trained populations, and its relevance remains unclear (Walker et al., 2016). For example, Benninger and coworkers found no significant differences in $\dot{V}$ O_2max_, workload, HR or respiratory rate between athletes who were tested with a blocked nose, a decongested nose, or received a nasal placebo spray during a stepwise maximal aerobic test (Benninger et al., 1992).

While most of these studies included relevant physiologic measures, such as $\dot{V}$ O_2max_ and HR, they found no detectable contribution of the nasal airway to performance breathing conditions. However, these studies lacked consistent measurements of nasal resistance and objective markers of exercise intensity, such as the blood lactate concentration. Furthermore, several studies used small samples with heterogeneity of sex distribution and fitness levels, thereby limiting the generalizability of the obtained results. To address the methodologic limitations, the present study investigates whether the nasal contribution during oronasal breathing affects performance in well-trained endurance athletes. Specifically, it aims to determine whether oronasal breathing, and especially in situations in which the nose is decongested, can: 1) increase performance, measured as time to exhaustion; and 2) influence the subjective ratings of exertion during incremental exercise to exhaustion. We hypothesize that allowing nasal airflow during oronasal breathing–particularly when the nasal passages are decongested–enhances the ventilatory efficiency and, thereby, modestly improves $\dot{V}$ O_2max_ and W_max_ compared to oral-only breathing, despite the primary limitations to maximal performance being cardiovascular and muscular in nature (Bassett and Howley, 2000).

## 2 Methodology

### 2.1 Study participants

Thirteen well-trained, male cyclists and triathletes in the age range of 19–48 years were recruited in the Gothenburg region of Sweden via contacts. One participant who completed only one of the three experimental trials before withdrawing due to a common cold was excluded from the final analysis. Thus, 12 participants aged (mean ± SD) 30.6 ± 8.7 years (height, 181.9 ± 5.4 cm; body weight, 75.8 ± 4.3 kg; percent fat mass, 14.4 ± 3.6; and $\dot{V}$ O_2max_, 67.2 ± 5.5 mL kg^-1^·min^-1^) completed all the study procedures. All the participants had prior experience in competitive cycling at the regional, national or elite level with a median of 10 years of competition history. Overall, 16% of the participants reported allergic rhinitis and 25% reported asthma with routine asthma medication. The study was conducted outside the pollen season in Sweden (January–March). All participants provided written informed consent prior to enrollment. The study was approved by the Swedish Ethical Review Authority (Dnr. 2020-03808) and registered at ClinicalTrials.gov (Identifier: NCT06480071) under the protocol titled *The Role of Nasal Breathing for Performance in Elite Athletes* (Sahlgrenska University Hospital, Protocol Record Dnr. 2020-03808).

### 2.2 Study overview

**FIGURE 1:**
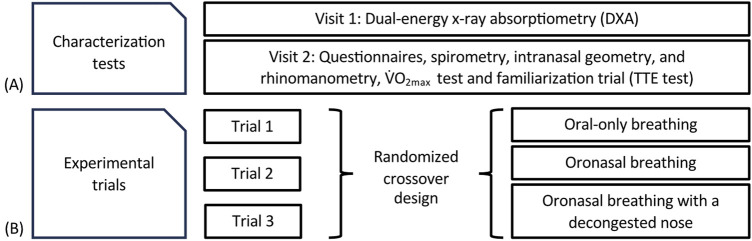
Flowchart of the study design and schematic of **(A)** the characterization tests; and **(B)** the experimental exercise trials. TTE; Time to exhaustion.

As illustrated in Figure 1, participants attended five separate visits to the exercise laboratory at the Center for Health and Performance (CHP), Department of Food and Nutrition and Sport Science (IKI), University of Gothenburg (GU). These visits comprised two characterization sessions and three experimental trials.

The study employed a novel design to evaluate nasal dynamics during oronasal breathing in endurance exercise. This was achieved by combining standardized cardiopulmonary exercise testing (CPET) with systematic nasal examinations across a range of exercise intensities, from low intensity up to $\dot{V}$ O_2max_. The two characterization sessions served several purposes. First, body composition was assessed, and $\dot{V}$ O_2max_ was determined. The latter was used to calculate each participant’s maximal workload (W_max_, 392 ± 38 W), which was used to individualize the experimental protocol. Participants also completed a familiarization trial in order to become accustomed with the maximal test procedures. In addition, a nasal examination was performed by an ear, nose, and throat (ENT) physician to screen for any significant anatomical abnormalities. The three experimental trials were conducted in randomized order, with participants serving as their own controls. The randomization sequence was generated in Microsoft Excel using the RAND function, with each condition assigned a numerical code and sorted to determine the trial order for each participant. Each participant completed the same individualized exercise protocol under the following three conditions.

(i) oral-only breathing (with the nose occluded using a nose clip);
(ii) oronasal breathing; and
(iii) oronasal breathing following nasal decongestion.

The minimum washout period between trials was 4 days, with a mean duration of 23 ± 14 days between the first and third experimental session. To ensure adherence to the assigned breathing routes, all participants wore a standard V2 face mask (Hans Rudolph Inc., United States). In the oral-only condition, nasal airflow was mechanically occluded using a nose clip positioned beneath the mask to prevent nasal breathing. In the oronasal condition, no such restriction was applied. Investigators continuously monitored participants throughout each test to verify breathing route adherence. Although the mask itself does not restrict nasal airflow, the combination of the nose clip in the oral-only condition and continuous visual monitoring minimized the risk of unintentional deviation. In the nasal decongestion trial, participants were administered oxymetazoline 0.5 mg/mL (Otrivin®; Haleon Denmark ApS, Copenhagen, Denmark), with two sprays in each nostril 10 min before exercise onset to reduce congestion of the nasal mucosa.

Variables related to CPET measurements collected during the experimental trials included: oxygen consumption ( $\dot{V}$ O_2_); carbon dioxide production ( $\dot{V}$ CO_2_); respiratory exchange ratio (RER); breathing frequency (BF); minute ventilation (VE); heart rate (HR); and capillary blood lactate and glucose (GLU) concentrations, as well as rating of perceived exertion (RPE, Borg scale 6–20) (Borg, 1970). Performance-related measures included power output (W) and time to exhaustion (seconds). Additional respiratory variables included ventilatory equivalents for oxygen and carbon dioxide ( $\dot{V}$ E/ $\dot{V}$ O_2_ and $\dot{V}$ E/ $\dot{V}$ CO_2_) and $\dot{V}$ O_2_/kg. Simultaneously, physiological examination variables were measured and included spirometry, intranasal geometry, and rhinomanometry.

Participants were instructed to refrain from alcohol and vigorous physical activity during the 24 h preceding each visit. Upon arrival at the CHP, height and nude body mass (BM) were measured (seca 764; seca GmbH, Hamburg, Germany), and hydration status was assessed via urine specific gravity (USG), with values ≤ 1.025 (Atago Co. Ltd., Tokyo, Japan) considered as indicative of euhydration. All exercise tests were performed on a cadence-independent cycle ergometer (LC7TT; Monark AB, Vansbro, Sweden) under standardized environmental conditions (ambient pressure, 759 ± 10 mmHg; temperature, 20.5°C ± 0.5°C; relative humidity, 31.7% ± 3.0%) (Vaisala PTU300; Vaisala Oyj, Vantaa, Finland). Gas exchange parameters ( $\dot{V}$ O_2_, $\dot{V}$ CO_2_) and HR were continuously recorded using the same metabolic measurement system across trials (Quark RMR/CPET; COSMED, Rome, Italy). Blood samples for lactate and GLU were collected before, during, and after each exercise test and analyzed using the Biosen C-Line system (EKF Diagnostics GmbH, Barleben, Germany). All items of equipment were calibrated prior to each trial according to the manufacturers’ instructions.

### 2.3 Characterization tests

At the first characterization visit (Figure 1), body composition was assessed following an overnight fast using dual-energy x-ray absorptiometry (DXA; GE Medical Systems, Madison, WI, United States). The accompanying enCore software (ver. 16.10) was used to calculate automatically the whole-body fat mass and fat-free mass. To standardize the pre-assessment conditions, participants were instructed to consume 500 mL of water upon waking and to use a low-intensity mode of transportation (e.g., walking or cycling with minimal effort) to the CHP laboratory.

At the second characterization visit, a multi-step nasal examination was performed prior to the experimental trials. First, the participants completed a nose-related questionnaire to assess potential nasal obstruction. Subsequently, an ENT specialist conducted an anterior rhinoscopic examination to identify any significant anatomical abnormalities, such as septal deviation, nasal polyps or signs of nasal inflammation.

The $\dot{V}$ O_2max_ was determined using a standardized CPET test, as described in detail previously (Pettersson et al., 2020). In brief, W_max_ was estimated by extrapolating the $\dot{V}$ O_2_ values from submaximal workloads to $\dot{V}$ O_2max_ using linear regression. Following the $\dot{V}$ O_2max_ test, participants rested for approximately 30 min before completing a familiarization trial. Each experimental trial lasted approximately 2 h and was conducted at the same time of day for each participant (e.g., all in the morning or afternoon). Participants were instructed to record their dietary intake on the day of the first trial and to replicate that dietary intake before each subsequent trial.

### 2.4 Experimental trials

**FIGURE 2:**
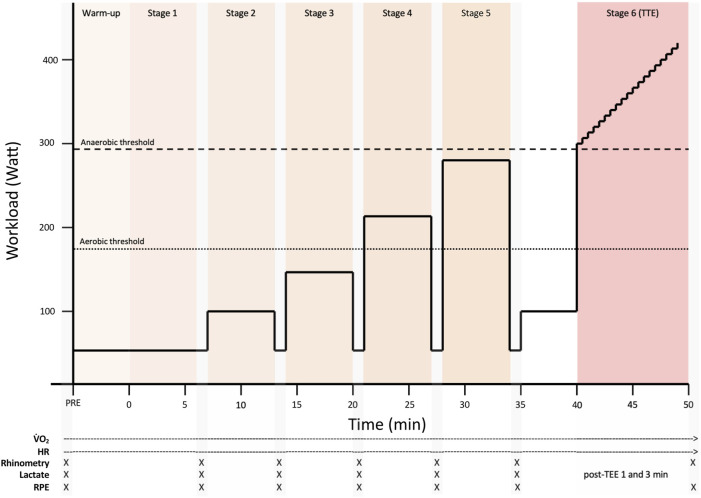
An overview of the experimental trial protocol. Each trial consisted of five 6-min submaximal, steady-state exercise stages followed by a sixth stage–an incremental maximal performance test to exhaustion. HR = Heart rate, RPE = Rating of perceived exertion, TTE = Time to exhaustion.

The experimental trial protocol is illustrated in Figure 2. Each test consisted of five 6-min submaximal, steady-state exercise stages followed by a sixth stage–an incremental maximal performance test to exhaustion.

The protocol was designed to span a physiologically relevant range from low to near-maximal intensities, covering workloads below, at, and above the first (LT1) and second (LT2) lactate thresholds, in accordance with established submaximal testing guidelines (Binder et al., 2008; Faude et al., 2009). After a warm-up at 50 W (50 rpm), stages 1 and 2 were set at 50 W and 100 W, both at 50 rpm, to provide low-intensity steady-state conditions for baseline respiratory and metabolic assessment. These stages also allowed all participants to perform identical absolute workloads, facilitating comparisons at matched submaximal levels. Given their low intensity, they fall within the range where a shift from nasal to oral breathing may occur (Niinimaa et al., 1980).

Stages 3–5 were performed at 40%, 58%, and 75% of each participant’s W_max_ (149 ± 18 W, 216 ± 26 W, and 280 ± 34 W) at 80 rpm, approximating LT1 and LT2 in trained endurance athletes while reflecting typical performance cadence. Following a 1-min recovery at 50 W and a 5-min re-warm-up at 40% W_max_, the final stage began at 80% W_max_ with a 1 W/6 s ramp at 90 rpm, consistent with validated protocols for inducing exhaustion while minimizing pacing effects (Bentley et al., 2007). CPET-related variables were measured continuously throughout each stage using a metabolic cart. For stages 1–5, gas exchange parameters, including $\dot{V}$ O_2_, $\dot{V}$ CO_2_, ventilation (VE), and respiratory frequency (RF), were averaged between minutes 3 and 4 of each stage. The RPE (Borg scale 6–20) was recorded during the following 1-min active rest (50 W), and fingertip capillary blood samples were taken to measure lactate levels. No verbal encouragement or time/physiologic feedback was given during the performance test, with the exception of cadence cues.

Nasal airway measurements were conducted immediately following each stage (Stages 1–6). Intranasal geometry was assessed using acoustic rhinometry, and nasal airflow resistance (NAR) was evaluated using anterior active rhinomanometry (A1 Acoustic Rhinometer and NR6 Rhinomanometer; GM Instruments, Irvine, Scotland). These assessments were also performed before trial. Rhinomanometry was conducted with a pressure probe inserted into one nostril and sealed with a foam plug. A transparent face mask covered the nose and mouth, measuring the airflow in the open nostril during nasal-only breathing with the mouth closed. The procedure was then repeated on the contralateral side. Data from three automatically approved breaths per side were used for the analysis. Acoustic rhinometry was performed with a nosepiece fitted to the nostril to prevent leakage. Each side was measured five times while the participant held their breath with a slightly open mouth.

During the final performance stage, gas exchange variables and heart rate values were averaged over the final minute of effort. The RPE was recorded immediately upon termination, and lactate was measured at 1 and 3 min post-exercise. Spirometry was conducted before each test using a nose clip (Spiro-SP TrueFlow, Spirare; Diagnostica AS, Oslo, Norway).

## 3 Statistical analyses

Descriptive statistics are presented as mean and standard deviations. Cohen’s d (d) was calculated as a standardized measure of effect size for pairwise comparisons. Data were analyzed with mixed models to account for the repeated measurements, with random effects for subject and breathing mode. For stage (of the incremental exercise test), we modeled the dependence among residuals (R-side effects) with a first-order autoregressive covariance structure, i.e., AR (1), in some cases with a heterogenous variance, i.e., ARH (1). We considered different types of covariance matrices (variance components, compound symmetry, unstructured, compound symmetry with separate parameters across levels) and selected the ones with the lowest Akaike information criterion where convergence was attained. All analyses were performed using SAS version 9.4 TS1M7 (SAS Institute, Cary, North Carolina, United States). A two-sided p-value <0.05 was considered statistically significant.

## 4 Results

The results of the three experimental trials, including performance-related variables, are shown in Table 1, and their associated effect size estimates (Cohen’s d) are detailed in Table 2. The values of W_max_ during the time-to-exhaustion test did not differ significantly (p > 0.05) between the breathing modes. The time to exhaustion was 2.8% shorter with oral-only breathing compared to oronasal breathing (d = −0.19), and 4.2% shorter compared to oronasal breathing with nasal decongestion (d = −0.28); however, these differences were not statistically significant (p > 0.05). No significant differences were observed between the three breathing modes for RPE, RF, $\dot{V}$ E, $\dot{V}$ O_2_, $\dot{V}$ CO_2_, RER, $\dot{V}$ E/ $\dot{V}$ O_2_, $\dot{V}$ E/ $\dot{V}$ CO_2_, ˙ $\dot{V}$ O_2_/kg BM or HR in the maximum exercise stage (Stage 6), as shown in Table 1. The mean capillary blood lactate concentrations were significantly lower for oral-only breathing than for both oronasal breathing conditions (with and without nasal decongestion) in the maximum exercise stage (Stage 6) and for the 1- and 3-min tests after time to exhaustion, as shown in Table 1.

**TABLE 1:** Cardiopulmonary, perceptual, metabolic, and performance responses across six incremental exercise stages and at 3 min post–time to exhaustion (TTE) in well-trained endurance athletes (N = 12), assessed under three experimental conditions: oral-only breathing (o), oronasal breathing (on), and oronasal breathing with a decongested nose (ond). Data are presented as mean (SD).

	Trial	Stage 1		Stage 2		Stage 3		Stage 4		Stage 5		TTE (stage 6)		3-min post TTE		p-value trial	p-value stage *trial
RPE	o	7.7	(0.8)	9	(1.7)	10.9	(1.8)	12.8	(1.5)	14.9	(1.1)	18.9	(0.7)				0.77
	on	7.5	(0.8)	9.3	(1.3)	11.6	(1.1)	12.8	(1.4)	14.8	(1.3)	18.8	(1.1)			0.74	
	ond	7.7	(1.1)	9.1	(1.2)	10.9	(1.2)	12.8	(1.1)	14.5	(1.2)	18.5	(1.6)				
RF	o	18.3	(4.0)	20.3	(3.4)	22.9	(2.5)	26.8	(3.3)	30.4	(5)	55.6	(7.8)				0.15
	on	21.1	(4.1)	20.4	(2.8)	23.4	(3.6)	28.2	(3.7)	32.8	(4.2)	55.7	(6.8)			0.20	
	ond	22.2	(5.5)	23.6	(8.3)	24.4	(3.2)	26.5	(3.2)	32.3	(4.6)	53.7	(9.4)				
$\dot{V}$ E	o	24.6	(3.3)	35.1	(3.6)	49.9	(8.4)	70.4	(11.1)	90.7	(14.1)	174	(18.4)				0.33
	on	25.4	(3.9)	35.6	(4.2)	50.8	(6.3)	70.4	(9.9)	93.1	(11.2)	181.9	(23.3)			**0.05**	
	ond	26.8	(3.9)	36.9	(3.8)	51.6	(7)	71.3	(8.5)	95.0	(11.8)	175.1	(20.7)				
$\dot{V}$ O_2_	o	981	(85)	1,468	(74)	2,110	(226)	2,947	(310)	3,664	(438)	4,969	(541)				0.11
	on	1,005	(71)	1,489	(94)	2,145	(236)	2,888	(331)	3,648	(404)	5,011	(521)			0.74	
	ond	981	(64)	1,540	(123)	2,118	(180)	2,916	(300)	3,662	(415)	4,912	(532)				
$\dot{V}$ CO_2_	o	823	(96)	1,228	(77)	1801	(191)	2,597	(266)	3,364	(379)	5,085	(516)				0.79
	on	830	(83)	1,243	(101)	1837	(207)	2,561	(296)	3,369	(368)	5,154	(547)			0.90	
	ond	859	(79)	1,289	(90)	1853	(168)	2,609	(271)	3,426	(401)	5,081	(514)				
RER	o	0.84	(0.06)	0.84	(0.03)	0.86	(0.04)	0.88	(0.04)	0.92	(0.04)	1.03	(0.04)				0.15
	on	0.82	(0.04)	0.83	(0.03)	0.86	(0.03)	0.89	(0.03)	0.92	(0.02)	1.03	(0.04)			0.06	
	ond	0.88	(0.06)	0.84	(0.03)	0.87	(0.03)	0.9	(0.04)	0.94	(0.04)	1.04	(0.03)				
$\dot{V}$ E/ $\dot{V}$ O_2_	o	23.9	(3.3)	22.9	(2.1)	22.9	(2.9)	23.3	(3)	24.2	(2.9)	34.4	(2.8)				0.27
	on	23.7	(2.4)	22.9	(1.9)	22.9	(1.6)	23.7	(2.4)	24.9	(1.8)	35.5	(2.9)			0.08	
	ond	25.7	(3.4)	22.9	(2.4)	23.5	(2.6)	23.9	(2.2)	25.3	(1.9)	35.0	(2.8)				
$\dot{V}$ E/ $\dot{V}$ CO_2_	o	28.4	(2.9)	27.4	(2.4)	26.8	(2.9)	26.4	(3.2)	26.4	(3.3)	33.6	(3.1)				0.46
	on	28.8	(2.8)	27.4	(2.3)	26.8	(2)	26.7	(2.7)	27	(2.4)	34.5	(2.8)			0.35	
	ond	29.3	(3.0)	27.3	(2.4)	26.9	(2.7)	26.6	(2.3)	27.1	(1.9)	33.7	(2.5)				
$\dot{V}$ O_2_/kg	o	13.0	(1.3)	19.4	(1.5)	27.9	(3)	38.9	(3.8)	48.4	(5.5)	65.6	(6.8)				0.15
	on	13.3	(1.6)	19.7	(1.6)	28.4	(3.1)	38.2	(4.3)	48.2	(5.5)	66.2	(6.6)			0.87	
	ond	13.0	(0.8)	20.4	(2.3)	28.1	(2.4)	38.6	(3.7)	48.5	(5.5)	65.0	(6.7)				
HR	o	78.1	(8.5)	93.1	(7.5)	111.6	(7.3)	131.2	(8.1)	150	(10.4)	181.4	(7.0)				0.82
	on	77.9	(11.1)	93.5	(9)	110.8	(6.9)	132.1	(8.8)	151.4	(10.7)	182.8	(9.0)			0.77	
	ond	77.5	(11.0)	92.1	(11.1)	110.4	(9.1)	130.7	(11.0)	150.2	(12.0)	181.2	(8.7)				
Lactate	o	1.1	(0.4)	1.1	(0.9)	0.9	(0.24)	1.1	(0.3)	2.3	(0.6)	9.1	(2.2)	8.6	(2.4)		**<0.005**
	on	1.1	(0.4)	0.9	(0.3)	1.0	(0.3)	1.4	(0.9)	2.3	(1.1)	9.8	(2.2)	9.6	(2.5)	**<0.005**	
	ond	1.2	(0.4)	0.9	(0.2)	0.9	(0.2)	1.4	(0.4)	2.4	(0.9)	9.8	(2.3)	9.6	(2.5)		

Abbreviations: RPE, Rating of Perceived Exertion; RF, Respiratory Frequency; $\dot{V}$ E, Ventilation (minute ventilation, total volume of air exhaled per minute); $\dot{V}$ O_2_, Oxygen Uptake (L·min^-1^); $\dot{V}$ CO_2_, Carbon Dioxide Production (L·min^-1^); RER, Respiratory Exchange Ratio; $\dot{V}$ E/ $\dot{V}$ O_2_, Ventilatory Equivalent for Oxygen; $\dot{V}$ E/ $\dot{V}$ CO_2_, Ventilatory Equivalent for Carbon Dioxide; $\dot{V}$ O_2_max, Relative Oxygen Uptake (mL·kg^-1^·min^-1^); HR, Heart Rate; TTE, Time to Exhaustion; Wmax, maximal workload. P-values < 0.05 are shown in bold. “Time” refers to the main effect of exercise stage; “Trial” refers to the main effect of breathing condition; and “Time × Trial” indicates the interaction between exercise stage and condition.

**TABLE 2:** Effect size estimates (Cohen’s d) for cardiopulmonary, perceptual, metabolic, and performance responses across six incremental exercise stages and at 3 min post time to exhaustion (TTE) in 12 well-trained endurance athletes (N = 12) under three experimental conditions: oral-only breathing (o), oronasal breathing (on), and oronasal breathing with a decongested nose (ond).

	Trial	Pre	Stage 1	Stage 2	Stage 3	Stage 4	Stage 5	TTE (stage 6)	3-min post TTE
RPE	o vs. on		0.24	−0.17	−0.45	0.06	0.07	0.10	
	o vs. ond		0.03	−0.06	0.00	0.06	0.36	0.29	
	on vs. ond		−0.17	0.14	0.59	0.00	0.27	0.20	
RF	o vs. on		−0.71	−0.02	−0.17	−0.38	−0.53	−0.01	
	o vs. ond		−0.81	−0.51	−0.50	0.10	−0.40	0.22	
	on vs. ond		−0.22	−0.52	−0.27	0.48	0.12	0.24	
$\dot{V}$ E	o vs. on		−0.22	−0.13	−0.11	0.00	−0.19	−0.37	
	o vs. ond		−0.59	−0.48	−0.21	−0.09	−0.33	−0.06	
	on vs. ond		−0.34	−0.32	−0.12	−0.10	−0.17	0.31	
$\dot{V}$ O_2_	o vs. on		−0.31	−0.25	−0.15	0.18	0.04	−0.08	
	o vs. ond		−0.01	−0.70	−0.04	0.10	0.00	0.11	
	on vs. ond		0.35	−0.46	0.13	−0.09	−0.03	0.19	
$\dot{V}$ CO_2_	o vs. on		−0.07	−0.17	−0.18	0.13	−0.01	−0.13	
	o vs. ond		−0.41	−0.72	−0.29	−0.05	−0.16	0.01	
	on vs. ond		−0.37	−0.48	−0.09	−0.17	−0.15	0.14	
RER	o vs. on		0.30	0.08	−0.04	−0.14	−0.18	−0.08	
	o vs. ond		−0.60	−0.03	−0.53	−0.33	−0.45	−0.27	
	on vs. ond		−1.10	−0.12	−0.63	−0.24	−0.35	−0.21	
$\dot{V}$ E/ $\dot{V}$ O_2_	o		0.05	0.02	−0.01	−0.16	−0.29	−0.40	
	o vs. ond		−0.54	0.00	−0.23	−0.22	−0.45	−0.21	
	on vs. ond		−0.66	−0.02	−0.29	−0.06	−0.22	0.20	
$\dot{V}$ E/ $\dot{V}$ CO_2_	o vs. on		−0.14	−0.01	0.00	−0.12	−0.21	−0.32	
	o vs. ond		−0.29	0.04	−0.04	−0.09	−0.26	−0.05	
	on vs. ond		−0.17	0.05	−0.05	0.04	−0.04	0.30	
$\dot{V}$ O_2_/kg	o vs. on		−0.25	−0.19	−0.16	0.17	0.02	−0.10	
	o vs. ond		−0.02	−0.53	−0.07	0.08	−0.03	0.08	
	on vs. ond		0.27	−0.37	0.11	−0.10	−0.05	0.18	
HR	o vs. on		0.02	−0.06	0.11	−0.10	−0.13	−0.17	
	o vs. ond		0.06	0.10	0.15	0.05	−0.01	0.03	
	on vs. ond		0.04	0.14	0.05	0.14	0.11	0.18	

**TABLE 2:** () Effect size estimates (Cohen’s d) for cardiopulmonary, perceptual, metabolic, and performance responses across six incremental exercise stages and at 3 min post time to exhaustion (TTE) in 12 well-trained endurance athletes (N = 12) under three experimental conditions: oral-only breathing (o), oronasal breathing (on), and oronasal breathing with a decongested nose (ond).

	Trial	Pre	Stage 1	Stage 2	Stage 3	Stage 4	Stage 5	TTE (stage 6)	3-min post TTE
Time to complete TTE (sec)	o vs. on							−0.19	
	o vs. ond							−0.28	
	on vs. ond							−0.10	
Watt	o vs. on							0.00	
	o vs. ond							0.05	
	on vs. ond							0.05	
Lactate	o vs. on	0.22	0.11	0.34	−0.56	−0.43	0.04	−0.32	−0.43
	o vs. ond	0.35	−0.31	0.32	−0.20	−0.67	−0.15	−0.31	−0.41
	on vs. ond	0.09	−0.39	−0.09	0.42	0.07	−0.14	0.01	0.02

Abbreviations: RPE, Rating of Perceived Exertion; RF, Respiratory Frequency; $\dot{V}$ E, Ventilation (total exhaled air per minute); $\dot{V}$ O_2_, Oxygen Uptake (L·min^-1^); $\dot{V}$ CO_2_, Carbon Dioxide Production (L·min^-1^); RER, Respiratory Exchange Ratio; $\dot{V}$ E/ $\dot{V}$ O_2_, Ventilatory Equivalent for Oxygen; $\dot{V}$ E/ $\dot{V}$ CO_2_, Ventilatory Equivalent for Carbon Dioxide; $\dot{V}$ O_2_/kg, Relative Oxygen Uptake (mL·kg^-1^·min^-1^); HR, Heart Rate; TTE, Time to Exhaustion; Wmax, maximal workload; Time at Wmax, duration at maximal workload.

**FIGURE 3:**
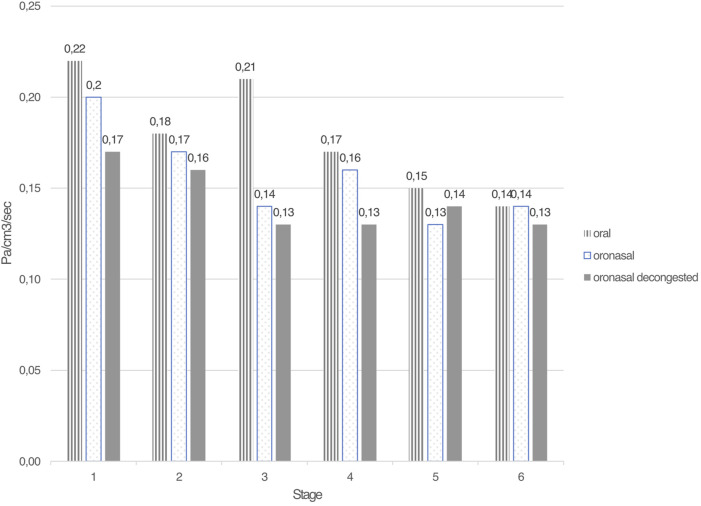
Mean nasal airway resistance (in Pa/cm^3^/sec) for the 6 test stages during oral-only, oronasal and oronasal decongested breathing.

The results for the mean NAR, mean nasal airflow, mean minimal cross-sectional area (MCA), and mean nasal volume are listed in Table 3 and Figure 3. There were no significant differences (p > 0.05) in the nasal function measures between the three breathing modes.

**TABLE 3:** Mean nasal airway resistance and mean nasal airflow, mean minimal cross-sectional area and mean nasal volume at 0–5 cm from the nostril at the 6 stages during: oral-only breathing (o), oronasal breathing (on) and oronasal breathing with a decongested nose (ond). Data are presented as mean (SD).

Trial	Stage	Nasal airway resistance (Pa/(cm^3^/s))		Nasal airflow (mL/s)		Minimal cross-sectional area (cm^2^)		Nasal volume at 0–5 cm from nostril (cm^3^)	
o	1	0.22	(0.13)	594	(179)	1.35	(0.20)	22.45	(7.08)
	2	0.18	(0.08)	844	(308)	1.39	(0.18)	23.12	(8.67)
	3	0.21	(0.12)	914	(365)	1.37	(0.19)	27.41	(8.17)
	4	0.17	(0.07)	1.021	(337)	1.34	(0.17)	29.44	(8.35)
	5	0.15	(0.09)	1.244	(409)	1.35	(0.30)	27.20	(9.02)
	6	0.14	(0.05)	1.211	(286)	1.31	(0.22)	23.53	(7.04)
on	1	0.20	(0.10)	744	(278)	1.37	(0.28)	23.53	(7.63)
	2	0.17	(0.05)	989	(356)	1.40	(0.26)	25.65	(10.67)
	3	0.14	(0.04)	1.176	(349)	1.38	(0.22)	25.21	(8.25)
	4	0.16	(0.08)	1.232	(282)	1.44	(0.25)	28.41	(5.83)
	5	0.13	(0.04)	1.268	(354)	1.33	(0.27)	29.49	(12.66)
	6	0.14	(0.05)	1.273	(310)	1.35	(0.24)	27.34	(7.56)
ond	1	0.17	(0.05)	984	(273)	1.39	(0.25)	35.12	(8.38)
	2	0.16	(0.05)	1.041	(353)	1.42	(0.26)	34.99	(12.63)
	3	0.13	(0.04)	1.221	(329)	1.41	(0.19)	33.45	(9.14)
	4	0.13	(0.05)	1.284	(368)	1.44	(0.15)	37.45	(12.60)
	5	0.14	(0.06)	1.225	(400)	1.39	(0.32)	34.10	(8.94)
	6	0.13	(0.06)	1.314	(477)	1.33	(0.19)	28.76	(10.75)

## 5 Discussion

This randomized, controlled, cross-over trial investigated the effects of oral-only (mouth) breathing versus oronasal breathing–with and without nasal decongestion–during a graded maximal exercise test. The outcomes included performance (power output and TTE), pulmonary gas exchange variables, blood lactate concentrations, HR values, and perceived exertion levels in well-trained, male endurance athletes. No significant differences were observed between the three breathing conditions during the incremental (submaximal) stages. However, performance during the final stage, i.e., the maximal test to exhaustion, was notably, albeit not significantly, lower during the oral-only condition compared to oronasal breathing, with or without nasal decongestion. In contrast, the mean capillary blood lactate concentrations were significantly lower with oral-only breathing compared to the oronasal breathing conditions. All participants reported a low subjective sensation of nasal obstruction on the VAS scale at baseline, exhibited normal spirometry before each test, and their levels of nasal airway resistance were reduced by more than 50% between the start and end of the three experimental tests, as expected (Table 3) (Forsyth et al., 1983).

Although $\dot{V}$ O_2max_ and W_max_ are primarily limited by cardiovascular and muscular factors in healthy, trained individuals (Bassett and Howley, 2000), the efficiency of the ventilatory system may still influence performance, particularly under conditions of maximal exertion. In addition to cardiovascular and muscular factors, respiratory limitations may arise during high-intensity exercise. At ≥85% of $\dot{V}$ O_2max_, in creased work of breathing (Wresp) can trigger a respiratory muscle metaboreflex, causing sympathetically mediated vasoconstriction in locomotor muscles and reduced perfusion (Harms et al., 1997; Dempsey et al., 2006). Large intrathoracic pressure swings may also impair venous return and stroke volume, affecting cardiac output and oxygen delivery (Sheel et al., 2001). While these mechanisms were not directly measured in the present study, they offer plausible explanations for subtle differences in time to exhaustion and lactate accumulation between breathing conditions. Our hypothesis–that allowing nasal airflow during oronasal breathing, especially when the nasal passages are decongested, improves ventilatory efficiency and, thereby, modestly enhances $\dot{V}$ O_2max_ and/or W_max_–is not supported by the results. However, it is grounded in seral plausible physiologic mechanisms. Nasal breathing facilitates the endogenous production and delivery of nitric oxide (NO), which is a vasodilator that is produced in the paranasal sinuses and that has been shown to improve ventilation-perfusion matching and pulmonary oxygen uptake (Lundberg et al., 1996). NO has been shown to regulate mucociliary clearance, as well as blood flow and oxygen exchange in the lungs (Lundberg, 2008). In addition, increased nasal airflow may reduce the anatomical dead space and the work of breathing, particularly when upper airway resistance is lowered through decongestion. Although this has not been extensively studied in athletic populations, the results from high-flow nasal cannula (HFNC) therapy in clinical settings suggests that enhanced nasal flow improves ventilatory efficiency by clearing the dead space and reducing the breathing effort (Nishimura, 2015). These mechanisms could theoretically influence the ventilatory equivalents ( $\dot{V}$ E/ $\dot{V}$ O_2_ and $\dot{V}$ E/ $\dot{V}$ CO_2_) and support more-efficient oxygen delivery during high-intensity exercise. However, in the present study, no significant differences in V̇E were observed between the breathing modes, indicating that increased nasal airflow does not enhance total ventilation under maximal load, or limited sensitivity of the current design. Future studies with larger cohorts and, where feasible, continuous or isolated nasal airflow measurements could help clarify the role of nasal breathing during high-intensity exercise.

Although not statistically significant, the 2.8% and 4.2% longer times to exhaustion observed during oronasal breathing, without and with nasal decongestion, respectively, compared to oral-only breathing, suggest a subtle performance benefit that is potentially meaningful in competitive settings where marginal gains are critical. The corresponding effect sizes (Cohen’s *d* = 0.19 and 0.28) were small, with the latter nearing the threshold for a moderate effect. Based on Cohen’s established guidelines (*d* ≈ 0.2 = small, 0.5 = moderate, 0.8 = large), such effects may still have practical relevance for elite athletes, for whom small improvements can influence the outcomes (Cohen, 1988). Further research is warranted to explore the ergogenic potentials of nasal decongestion strategies across different exercise modes, intensities, and durations.

In addition to $\dot{V}$ O_2max_, the blood lactate concentration is commonly used in exercise physiology as a marker of metabolic stress, training intensity, and endurance performance capacity in athletes (Seiler, 2010). In the present study, the significantly lower mean capillary blood lactate concentration observed following oral-only breathing during the maximal test suggests that a single, low-resistance airway is metabolically more efficient than parallel airflow through both the nasal and oral passages, as occurs in oronasal breathing. In a recent randomized, cross-over study that investigated low-intensity exercise, the capillary blood lactate levels were significantly lower following nose-only breathing than following oronasal breathing (Rappelt et al., 2023). This finding suggests that nasal breathing confers metabolic advantages during low-intensity exercise, a context in which blood lactate concentrations are typically maintained at a steady state. However, as exercise intensity increases, a physiologic breakpoint (known as the *lactate threshold*) is reached, beyond which the lactate levels increase exponentially, reflecting a shift towards greater anaerobic metabolism. A change in blood lactate concentration during submaximal exercise can indicate a shift in substrate utilization. To explore this, we measured $\dot{V}$ O_2_ consumption and $\dot{V}$ CO_2_ production, using the RER as a marker of substrate preference. As expected, the RER increased systematically with rising exercise intensity (p < 0.0001), reflecting a greater reliance on carbohydrate oxidation at higher workloads. No significant main effect of the trial was observed (p = 0.06), indicating that the breathing condition (oral-only, oronasal, or decongested oronasal breathing) does not significantly alter the RER. In addition, no significant trial × time interaction was found (p = 0.15), suggesting that the rate of increase of RER over time remains consistent across different breathing conditions. It is important to note that blood lactate levels are influenced not only by the rate of production, primarily via glycolysis, but also by the rate of clearance, as lactate can be oxidized and used as a substrate by skeletal muscles and other tissues (Bartoloni et al., 2024). The lactate threshold is, therefore, highly individual and closely related to an athlete’s training status and oxidative capacity. Thus, the 3%–4% reduction in work duration is a plausible explanation for the lower lactate levels observed at the time of and following maximum exertion.

### 5.1 Strengths and limitations

A key strength of the present study is the inclusion of capillary blood lactate level as an objective marker of exercise intensity, complementing other physiologic and ventilatory parameters. The use of time to exhaustion and power output as performance outcomes, alongside HR, ventilatory equivalents, and perceived exertion, provide a comprehensive physiologic profile. A limitation of this study is the absence of arterial oxygen pressure (PaO_2_) or peripheral oxygen saturation (SpO_2_) measurements during exercise. Consequently, it is not possible to determine whether participants experienced exercise-induced hypoxemia (EIH), commonly defined as a ≥10 mmHg drop in PaO_2_ and a ≥5% reduction in arterial oxygen saturation (SaO_2_) during intense exercise compared to resting values (Dempsey et al., 1984). Given the high aerobic capacity of the participants, EIH cannot be ruled out as a potential factor influencing oxygen delivery and performance. Another notable strength is the relatively homogeneous group of participants, in that all the subjects were well-trained male cyclists, highly experienced in their sport and familiar with cycle ergometry testing. This reduced the inter-individual variability related to sex, fitness level, sport-specific adaptations, and testing familiarity, which are factors that could have influenced the results in previous studies. Although participants were uniformly well-trained and familiar with ergometer cycling, including both cyclists and triathletes may have introduced minor sport-specific differences (e.g., ventilatory mechanics; (Bentley et al., 2002)). However, the within-subject crossover design minimizes the impact of such variation on comparisons between breathing conditions. The present study also stands out for its detailed and repeated measurements of nasal airway function using both rhinomanometry and acoustic rhinometry, not only at rest but also during each incremental stage and post-exhaustion. This offers novel insights into the dynamic behavior of the nasal airflow during exercise. In line with earlier studies (Forsyth et al., 1983; Syabbalo et al., 1985), our findings confirm that there is a progressive reduction in nasal airway resistance during physical activity. There were no statistically significant differences in nasal resistance between the breathing modes, although a more-rapid reduction in resistance was observed under the decongested condition.

One limitation is that the experimental exercise trial used in this study has not been previously validated. However, the protocol was specifically designed to enable reliable and valid integration of cardiopulmonary exercise testing with repeated nasal airway assessments. Submaximal workloads were strategically selected to span a physiologic range that spanned from below to above the aerobic and anaerobic thresholds, while still ensuring steady-state conditions (Binder et al., 2008), making it appropriate for the dual purposes of this investigation. In addition to protocol validation, methodological constraints related to breathing route verification should also be acknowledged. A limitation is the inability to objectively verify precise breathing routes, particularly during oronasal trials. Although visual monitoring was used and nasal airflow was blocked in the oral-only condition, brief or subtle deviations may have gone undetected. While physical restrictions have been applied in low-intensity settings (Rappelt et al., 2023), real-time nasal airflow monitoring during high-intensity exercise remains technically and practically challenging.

## 6 Conclusion

This randomized, controlled cross-over trial shows that the time to exhaustion is 2.8% and 4.2% longer during oronasal breathing and decongested oronasal breathing, respectively, as compared with oral-only breathing. Although these differences did not reach statistical significance, effect size estimates (Cohen’s *d* = 0.19–0.28) suggest a small to potentially meaningful physiological impact. Oral-only breathing was associated with lower post-exercise blood lactate concentrations; however, this could reflect, at least partly, the shorter exercise duration, implying that reduced metabolic strain may result from less total work performed rather than from the breathing route itself.

Nevertheless, the consistent association between exclusive oral breathing and lower lactate suggests that breathing route may still influence lactate accumulation, warranting further investigation. Taken together, these findings imply that minimizing nasal airway resistance supports endurance performance, while exclusive oral breathing may alter metabolic responses. Future studies should explore nasal airflow dynamics and decongestion strategies across different exercise modalities and athlete populations.

## Data Availability

The raw data supporting the conclusions of this article will be made available by the authors, without undue reservation.

## References

[B1] Bartoloni B. Mannelli M. Gamberi T. Fiaschi T. (2024). The multiple roles of lactate in the skeletal muscle. Cells 13 (14), 1177. 10.3390/cells13141177 39056759 PMC11274880

[B2] Bassett D. R. Jr. Howley E. T. (2000). Limiting factors for maximum oxygen uptake and determinants of endurance performance. Med. Sci. Sports Exerc 32 (1), 70–84. 10.1097/00005768-200001000-00012 10647532

[B3] Benninger M. S. Sarpa J. R. Ansari T. Ward J. (1992). Nasal patency, aerobic capacity, and athletic performance. Otolaryngol. Head. Neck Surg. 107 (1), 101–104. 10.1177/019459989210700116 1382258

[B4] Bentley D. J. Millet G. P. Vleck V. E. McNaughton L. R. (2002). Specific aspects of contemporary triathlon: implications for physiological analysis and performance. Sports Med. 32 (6), 345–359. 10.2165/00007256-200232060-00001 11980499

[B5] Bentley D. J. Newell J. Bishop D. (2007). Incremental exercise test design and analysis: implications for performance diagnostics in endurance athletes. Sports Med. 37 (7), 575–586. 10.2165/00007256-200737070-00002 17595153

[B6] Binder R. K. Wonisch M. Corra U. Cohen-Solal A. Vanhees L. Saner H. (2008). Methodological approach to the first and second lactate threshold in incremental cardiopulmonary exercise testing. Eur. J. Cardiovasc Prev. Rehabil. 15 (6), 726–734. 10.1097/HJR.0b013e328304fed4 19050438

[B7] Borg G. (1970). Physical training. 3. Perceived exertion in physical work. Lakartidningen 67 (40), 4548–4557. 5477775

[B8] Cohen J. (1988). Statistical power analysis for the behavioral sciences. New York, NY: Routledge. 2nd ed.

[B9] Dempsey J. A. Hanson P. G. Henderson K. S. (1984). Exercise-induced arterial hypoxaemia in healthy human subjects at sea level. J. Physiol. 355, 161–175. 10.1113/jphysiol.1984.sp015412 6436475 PMC1193484

[B10] Dempsey J. A. Romer L. Rodman J. Miller J. Smith C. (2006). Consequences of exercise-induced respiratory muscle work. Respir. Physiol. Neurobiol. 151 (2-3), 242–250. 10.1016/j.resp.2005.12.015 16616716

[B11] Dinardi R. R. Ferreira C. H. S. Silveira G. S. de Araujo Silva V. E. da Cunha Ibiapina C. de Andrade C. R. (2021). Does the external nasal dilator strip help in sports activity? A systematic review and meta-analysis. Eur. Arch. Otorhinolaryngol. 278 (5), 1307–1320. 10.1007/s00405-020-06202-5 32683573

[B12] Faude O. Kindermann W. Meyer T. (2009). Lactate threshold concepts: how valid are they? Sports Med. 39 (6), 469–490. 10.2165/00007256-200939060-00003 19453206

[B13] Forsyth R. D. Cole P. Shephard R. J. (1983). Exercise and nasal patency. J. Appl. Physiol. Respir. Environ. Exerc Physiol. 55 (3), 860–865. 10.1152/jappl.1983.55.3.860 6629922

[B14] Haight J. S. Cole P. (1983). The site and function of the nasal valve. Laryngoscope 93 (1), 49–55. 10.1288/00005537-198301000-00009 6823174

[B15] Harms C. A. Babcock M. A. McClaran S. R. Pegelow D. F. Nickele G. A. Nelson W. B. (1997). Respiratory muscle work compromises leg blood flow during maximal exercise. J. Appl. Physiol. 82 (5), 1573–1583. 10.1152/jappl.1997.82.5.1573 9134907

[B16] LaComb C. Lee S.-P. Young J. Navalta J. (2017). Oral versus nasal breathing during moderate to high intensity submaximal aerobic exercise. Int. J. Kinesiol. Sports Sci. 5, 8. 10.7575//aiac.ijkss.v.5n.1p.8

[B17] Lundberg J. O. (2008). Nitric oxide and the paranasal sinuses. Anat. Rec. Hob. 291 (11), 1479–1484. 10.1002/ar.20782 18951492

[B18] Lundberg J. O. Settergren G. Gelinder S. Lundberg J. M. Alving K. Weitzberg E. (1996). Inhalation of nasally derived nitric oxide modulates pulmonary function in humans. Acta Physiol. Scand. 158 (4), 343–347. 10.1046/j.1365-201X.1996.557321000.x 8971255

[B19] Meir R. Zhao G. G. Zhou S. Beavers R. Davie A. (2014). The acute effect of mouth only breathing on time to completion, heart rate, rate of perceived exertion, blood lactate, and ventilatory measures during a high-intensity shuttle run sequence. J. Strength Cond. Res. 28 (4), 950–957. 10.1519/JSC.0000000000000246 24077371

[B20] Niinimaa V. Cole P. Mintz S. Shephard R. J. (1980). The switching point from nasal to oronasal breathing. Respir. Physiol. 42 (1), 61–71. 10.1016/0034-5687(80)90104-8 7444224

[B21] Niinimaa V. Cole P. Mintz S. Shephard R. J. (1981). Oronasal distribution of respiratory airflow. Respir. Physiol. 43 (1), 69–75. 10.1016/0034-5687(81)90089-x 7244427

[B22] Nishimura M. (2015). High-flow nasal cannula oxygen therapy in adults. J. Intensive Care 3 (1), 15. 10.1186/s40560-015-0084-5 25866645 PMC4393594

[B23] Pettersson S. Ahnoff M. Edin F. Lingstrom P. Simark Mattsson C. Andersson-Hall U. (2020). A hydrogel drink with high fructose content generates higher exogenous carbohydrate oxidation and lower dental biofilm pH compared to two other, commercially available, carbohydrate sports drinks. Front. Nutr. 7, 88. 10.3389/fnut.2020.00088 32596251 PMC7303329

[B24] Rappelt L. Held S. Wiedenmann T. Deutsch J. P. Hochstrate J. Wicker P. (2023). Restricted nasal-only breathing during self-selected low intensity training does not affect training intensity distribution. Front. Physiol. 14, 1134778. 10.3389/fphys.2023.1134778 37153227 PMC10156973

[B25] Recinto C. Efthemeou T. Boffelli P. T. Navalta J. W. (2017). Effects of nasal or oral breathing on anaerobic power output and metabolic responses. Int. J. Exerc Sci. 10 (4), 506–514. 10.70252/EHDR7442 28674596 PMC5466403

[B26] Seiler S. (2010). What is best practice for training intensity and duration distribution in endurance athletes? Int. J. Sports Physiol. Perform. 5 (3), 276–291. 10.1123/ijspp.5.3.276 20861519

[B27] Sheel A. W. Derchak P. A. Morgan B. J. Pegelow D. F. Jacques A. J. Dempsey J. A. (2001). Fatiguing inspiratory muscle work causes reflex reduction in resting leg blood flow in humans. J. Physiol. 537 (Pt 1), 277–289. 10.1111/j.1469-7793.2001.0277k.x 11711580 PMC2278925

[B28] Syabbalo N. C. Bundgaard A. Widdicombe J. G. (1985). Effects of exercise on nasal airflow resistance in healthy subjects and in patients with asthma and rhinitis. Bull. Eur. Physiopathol. Respir. 21 (6), 507–513. 4074956

[B29] Walker A. Surda P. Rossiter M. Little S. (2016). Nasal function and dysfunction in exercise. J. Laryngol. Otol. 130 (5), 431–434. 10.1017/S0022215116000128 27095550

